# Qualitative process evaluation of a disability-inclusive ultra-poor graduation programme in Uganda

**DOI:** 10.4102/ajod.v13i0.1487

**Published:** 2024-11-11

**Authors:** Anthony Mugeere, Tom Shakespeare, Mark T. Carew

**Affiliations:** 1 School of Social Sciences, Makerere University, Kampala, Uganda; 2 International Centre for Evidence in Disability, London School of Hygiene and Tropical Medicine, London, United Kingdom

**Keywords:** poverty, livelihoods, disability, intervention, inclusive, development

## Abstract

**Background:**

There is a paucity of evidence regarding what works to help persons with disabilities escape the trap of poverty. To address extreme poverty among the general population, poverty graduation approaches have gained popularity. These programmes combine direct livelihood assistance (e.g. provision of assets) with wider support given to individuals (e.g. skill development). However, these interventions have rarely been adapted to be disability-inclusive.

**Objectives:**

The present research is a qualitative process evaluation of a disability-inclusive poverty graduation intervention, implemented in Uganda from 2020 to 2022. The study focusses on contextual influences on the intervention and mechanisms of impact according to the perspectives of implementers and intervention recipients, with a complementary analysis of structures and resources used to deliver the intervention derived from a desk-based review of programme reports.

**Method:**

In all, 15 implementers and 23 persons with disabilities who received the intervention were interviewed using semi-structured interviews. Interview data underwent framework analysis.

**Results:**

National infection prevention measures and loss of intervention funding associated with coronavirus disease 2019 (COVID-19) were identified as contextual influences on the intervention. Respondents highlighted increases in social empowerment and positive changes in societal attitudes to disability as routes through which the intervention had a positive impact. However, instances of jealousy from community members not receiving the intervention were also an unintended consequence.

**Conclusion:**

Results are discussed in terms of practical implications for delivering similar interventions in other contexts.

**Contribution:**

This study contributes new knowledge about the key factors that influenced the effectiveness of a disability-inclusive poverty graduation intervention.

Persons with disabilities are often described as the largest global minority group, comprising approximately 16% of the global population (World Health Organization 2022), and of whom an estimated 80% reside in low- and middle-income countries (World Health Organization 2011). There is strong evidence for the link between disability and increased poverty (Banks, Kuper & Polack 2017a), whereby poverty contributes to incidences of disability (e.g. malnutrition) and disability pushes individuals into poverty through consequent barriers, such as difficulties obtaining employment (Groce et al. 2011; Mitra, Posarac & Vick 2013). A recent study conducted by Pinilla-Roncancio and Alkire (2020) across five countries highlighted that not only were persons with disabilities and their families more likely to be facing severe multidimensional poverty, but that they were also experiencing greater levels of destitution in most of the countries studied. Poverty alleviation is a key focus of the 2030 Sustainable Development Goals (SDGs) which unlike the preceding Millennium Development Goals (MDGs) include an explicit focus on persons with disabilities as a group likely to experience marginalisation, encapsulated by the overarching aim of the SDGs to ‘leave no-one behind’ (Wei et al. 2023).

Despite this important objective, there has been markedly little attention to what may work to bring persons with disabilities and their families out of poverty, including how persons with disabilities can be effectively included in mainstream poverty alleviation programmes. A systematic review by Hunt et al. (2022) identified a lack of evidence concerning disability-inclusive livelihoods interventions, including an absence of robust impact evaluations. As such, while the impetus to alleviate poverty among persons with disabilities and their families is clear, how best to do this remains muddied. To address extreme poverty among the general population, poverty graduation approaches have gained popularity. Originally designed by the non-governmental organisation (NGO) BRAC (Matin, Rabbani & Sulaiman 2008) the ‘Ultra-Poor Graduation Programme’ (UPG) combines direct provision of livelihood support (e.g. asset transfers) for immediate needs with a wider programme of support and skill development designed to help the poorest households build productive and sustainable livelihoods, and permanently escape poverty. The results of six randomised trials with over 10 000 participants from Ethiopia, Ghana, Honduras, India, Pakistan and Peru suggest that poverty graduation approaches are effective for the general population of the ultra-poor (Banerjee et al. 2015). Specifically, the interventions were found to produce significant and cost-effective impacts on the primary metric of household consumption, as well as household assets, food security and income, the majority of which persisted 1-year post-intervention delivery. Gains were also shown for measures of wellbeing. Taken together, the results highlight the promise of the poverty graduation approach for those living in extreme hardship, including persons with disabilities.

However, to realise this promise, disability-inclusive poverty graduation programmes need to address the structural and multi-faceted barriers that persons with disabilities and their families face. For instance, many persons with disabilities face challenges in navigating their environments and require access to quality assistive devices or infrastructure adaptations before they can access services or engage in livelihood activities (Van Pletzen, Kabaso & Lorenzo 2021). Moreover, poverty alleviation programmes must account for how access and impact may be influenced by other demographic characteristics persons with disabilities possess, such as gender and impairment type (Banks et al. 2017b), as well as contextual influences specific to the intervention setting. Previous impact evaluations of BRAC’s UPG programme have not included persons with disabilities (Kipchumba et al. 2024). Thus, there persists a dearth of evidence about the extent to which the poverty graduation approach can be adapted to cater to the needs of persons with disabilities and their families. Equally important to understanding how well the intervention worked is comprehending why it did so, including context specific influences on its impact. Achieving this aim necessitates a process evaluation.

The present study is part of a wider mixed methods process evaluation of the Disability Inclusive Graduation Programme (DIG) implemented in Uganda from December 2020 to June 2022. This process evaluation is taking place alongside a cluster randomised control trial (cRCT) (see Kipchumba et al. 2024) as part of a comprehensive evaluation of DIG carried out by the Programme for Evidence to Inform Disability Action (PENDA) project and funded by the United Kingdom Foreign, Commonwealth & Development Office. Specifically, the present study is a qualitative process evaluation which explores contextual influences on intervention delivery and impact and mechanisms of impact according to the perspectives of implementers and intervention recipients.

## Research methods and design

### Study setting

The DIG programme was implemented within three districts located in Northern Uganda (Gulu, Nwoya and Oyam) and one in Western Uganda (Kiryandongo) by BRAC in partnership with Humanity and Inclusion (HI), a disability NGO, and the National Union of Women with Disabilities of Uganda (NUWODU) – an organisation of persons with disabilities. Its main aim is poverty reduction and economic empowerment. Within the DIG programme, the unit of participation was the household. Households were considered eligible if they met three of five criteria: (1) having a person with disability, (2) being a female-headed household or dependent on earnings from a female member of the household, (3) having children who are out of school, (4) poor housing conditions (floor, roof and wall), and/or (5) low productive asset endowment assessed through an identification survey (Kipchumba et al. 2024). For the impact evaluation, clusters were formed comprising aggregates of households (range: 10–75 households) and random allocation undertaken. A total of 96 clusters were assigned to the intervention (*N* = 2898 households) and the remaining 89 (*N* = 2402 households) to the control arm (Kipchumba et al. 2024). Control households do not receive the DIG programme, but have access to existing BRAC services.

### Design and procedure

Intervention implementers and recipients were interviewed by a team of four academic researchers experienced in qualitative research data collection and analysis. The research team was based in Uganda’s capital city (Kampala) and conducted face-to-face, telephone or online interviews with respondents selected for the process evaluation in order to comply with national coronavirus disease 2019 (COVID-19) Standard Operating Procedures (SoPs). Each interview lasted an average of an hour. All interviews were audio recorded and later transcribed by the research team.

### Respondents

A total of 38 semi-structured interviews were conducted, comprising 23 interviews with intervention recipients with disabilities and 15 informants based in Uganda who had been involved in the delivery of the DIG programme. The evaluation team decided that 38 was an appropriate sample size after realising that no new data was emerging from additional interviews with each group. Eligible intervention recipient respondents were purposively selected to obtain a diverse sample based on their demographics (primarily age, gender and impairment type), obtained by the research team from lists provided by the implementation team. The key informants for this evaluation comprised individuals who were BRAC staff or partners at the time of the programme implementation, and who were purposively selected by the programme management based on their knowledge and participation in the implementation of its activities. These comprised 11 key informants from BRAC, one from HI and three from the NUWODU.

Table 1 describes the main characteristics of respondents.

**TABLE 1:** Respondent characteristics.

Participant type	Description	*N*	Gender (*n*)		Location	*n*
			Male	Female		
Intervention recipients	Persons with disabilities	23	6	17	-	-
					Gulu	7
					Kiryandongo	5
					Nwoya	5
					Oyam	6
Implementers	BRAC staff or partner organisation staff	15	6	9	-	-
					Gulu	3
					Kiryandongo	6
					Nwoya	4
					Oyam	2

Of the intervention recipients interviewed, 14 had varying forms of physical disabilities, 6 had visual impairments and 3 had hearing impairments. For hearing impaired participants, interviews were carried out by research assistants who were proficient in Uganda Sign Language (USL) who recorded and later translated the data into English for analysis. Information sheets were read aloud to participants who had visual disabilities or who were illiterate. In addition, the intervention recipients who could not speak and understand English were interviewed in Luganda and Luo, and the data translated and transcribed in English for analysis.

### Data analysis

Data analysis of qualitative interviews used the Framework Analysis Approach (FAA) (Ritchie et al. 2003). This entailed familiarisation with the data, theme identification and interpretation of the data. After transcribing the interviews, the transcripts were read and re-read before constructing themes based on the specific objectives of the process evaluation.

By and large, the framework analysis enabled the creation of a new structure for the data (rather than the full original accounts given by the respondents) that the research team found quite helpful in summarising and/or reducing the data in a way that supported answering the objectives of the evaluation process. During the data analysis process, the study team selected and included specific quotes in the results that were illustrative of the identified themes (Mugeere, Oporia & Kobusingye 2022).

This analysis was complemented by a desk review of reports published on the DIG intervention by the implementation team, in particular a detailed close-out report (BRAC 2022). These documents were used to gain insight into the structures and resources used to deliver the intervention.

### Ethical considerations

Ethical approval was obtained from both the ethics committee of the London School of Hygiene and Tropical Medicine (Reference: 22619/RR/21198) and the Mildmay Uganda Research Ethics Committee (Reference: 0604–2020), with a research permit obtained from the Uganda National Council for Science and Technology (UNCST) (Reference: SS529ES). All data were kept confidential. Written and recorded informed consent was obtained from every participant prior to any interview.

## Findings

The study findings presented in this paper fall into three areas: structures and resources used to deliver the intervention, contextual factors that influenced the intervention and the mechanisms of impact through which the intervention operated.

### Structures and resources used to deliver the intervention

Participants were identified via household surveys in the target four districts by project staff using a digital survey tool. A total of 2700 households were enrolled into the intervention, of whom 454 were households with persons with disabilities. Among these households, a single individual within each household was designated as the main recipient of the training and assets (Kipchumba et al. 2024). A person with a disability was designated as the main recipient of the training and assets in half of the households with members with disabilities. The key intervention areas for the programme comprised four pillars namely: livelihood promotion, financial inclusion, social protection and social empowerment.

#### Livelihood promotion

Under the DIG programme, participants were able to choose a main and subsidiary asset from a range of prespecified options including different livestock (e.g. cows, goats, poultry), crops or trade support (specify). Most households (*N* = 1727) selected goats as their primary asset and poultry as their subsidiary asset (*N* = 1330) (BRAC 2022). The initial value of the assets given to each household was approximately 300 USD. The main recipient of the project then received a 3-day classroom training from project staff on how to set up their enterprise. In the case where these main recipients were persons with disabilities, assets were delivered directly to participant households and not collected from village centres as was the case with other recipients.

#### Financial inclusion

All project recipients with disabilities receive classroom trainings on financial literacy, aimed at building household capacity for financial management. Village Saving and Loans Association (VSLA) groups were formed to support the empowerment of persons with disabilities involved in the project, supported by an initial 2-days training. A total of 138 VSLA groups were formed, in which 209 persons with disabilities took leadership positions (BRAC 2022). The VSLA helped group members to make informed investment decisions and also become more connected within their community.

#### Social protection

All 2700 households received a monthly consumption stipend of approximately USD 18 in local currency, through a mobile money platform, and cash withdrawals facilitated by the VSLA. Emergency health subsidies were also offered to households on a needs basis, through Community Health Promotion project staff present in VSLA meetings.

All persons with disabilities in the 454 households with such household members also received rehabilitation and psychosocial assessments from project rehabilitation staff (i.e., occupational therapists, physiotherapists and psychosocial workers). Within these households, 214 persons were assessed as needing assistive devices. Assistive device procurement was done with the support of Humanity & Inclusion and participants were given either pre-fabricated devices or those that were custom-made depending on their need. Occupational therapists also carried out home and asset house (e.g. goat pen) assessments with participants with disabilities and worked with local builders to implement required adaptations (e.g. modifying doorways, building ramps). Environmental adaptations were carried out in 164 households with household members with disabilities. Additionally, 193 people with disability received rehabilitation support (e.g. treatment plan development), 154 received occupational therapy support (e.g. self-care and independent transfer training) and 156 received physiotherapy. Furthermore, 212 persons with disability also received psychosocial support from project psychosocial workers on a quarterly basis. Occupational therapy, physiotherapy and psychosocial support were generally provided on a quarterly basis (BRAC 2022).

#### Social empowerment

The project also formed Village Poverty Reduction Committees (VPRCs) as a way of linking participants to community initiatives aimed at improving their well-being. These VPRCs comprised volunteers from the community, who as part of their roles engaged men and women from participating households to promote positive norms around disability inclusion and gender equality (BRAC 2022). A total of 108 VPRCs were formed, in which 92 persons with disabilities were a committee member. The project also established peer support groups at village level, in which group members would discuss issues affecting persons with disabilities in the community (e.g. access to justice) and trained paralegals to support persons with disabilities to access justice, as this was deemed to be an obstacle by many ultra-poor people and persons with disabilities. Life-skills training was also provided to all recipients of the DIG programme, focussed on creating knowledge on health and social issues (e.g. gender empowerment, family planning, nutrition). Activities under this pillar were supported by an earlier mapping of access barriers and support services within target districts.

### The context of the intervention

This process evaluation identified two salient contextual factors that impacted implementation, both of which were precipitated by the COVID-19 pandemic: national infection prevention and control measures and loss of programme funding.

#### National infection prevention and control measures

The implementation of DIG was greatly affected by the outbreak of the COVID-19 pandemic, which entailed national compliance with infection prevention and control SoPs. These brought to a halt intervention components that involved physical mobilisation and movement of programme staff to hold face-to-face meetings with intervention recipients. The pandemic also limited the movement of intervention recipients to attend meetings and other engagements at the Programme headquarters. The situation was compounded by the lockdowns that were imposed at various stages of the pandemic as explained by one of the programme officers interviewed:

‘The COVID-19 pandemic was such a challenge to us. When we were put under lock down, the key question was: how were we going to handle the situation? How would our intervention recipients fare under such situations since we couldn’t access all of them as and when we needed to do so. This necessitated thinking outside the box and we did exactly that.’ (BRAC Programme Officer, Gulu district, Northern Uganda)

Although medical and other ‘essential’ workers were given special permission to travel and provide services during the lockdown, there were limitations on the number of individuals allowed to use personal cars at a particular time. Accordingly, most cars could only carry half their capacity which affected the number of programme staff who provided services (e.g. physiotherapy) to the intervention recipients as explained by one of the project staff interviewed:

‘If the car was designed to carry eight people, this time we did half which somehow affected teamwork. Instead of going as a bigger team, we found ourselves going lesser. These changes had to be made and they really affected us negatively.’ (BRAC Programme Officer, Kampala Metropolitan region)

Consequently, the COVID-19 pandemic inevitably reduced the level of direct contact between the programme staff and the intervention recipients. Given that some of the activities such as physiotherapy and other rehabilitation activities are essentially ‘hands on’, the pandemic literally reduced the number of hands provided by the programme staff. Even when a few could use individual transport such as motorcycles and other means of transport to reach some programme intervention recipients in dire need of such services, there was often little time to provide the services. Worse still, some programme staff were highly stigmatised by communities as they went about their daily duties – a point highlighted by another project staff member:

‘As we moved around the villages and trading centres, people were saying that we were the ones bringing COVID-19 to them, so it was also a bit negative. Against that background, there was nothing much we were able to change. It was a very difficult situation for us.’ (BRAC Project officer, Oyam district, Northern Uganda)

Although there was no radical overhaul of the programme design and implementation in response to COVID-19, the team was forced to expand and modify the implementation approaches and strategies. For instance, radio shows were conducted containing messaging about disability inclusion in national COVID-19 response as part of the overall strategy to scale up sensitisation campaigns which had been hitherto on a face-to-face basis – a view explained by one of the key informants interviewed:

‘During the COVID-19 period, we were sensitizing the people about disability and inclusion in the task force when they are making decisions because you know people were getting special development programs from different partners and we were letting them know that persons with disabilities also should be considered. We were also advocating that at least on the task force there should be a person with disability to be able to speak from within. So, we emphasised that whenever they were programming, they had to understand that there is a person with disability in the community.’ (BRAC Programme Officer, Kiryandongo district, Western Uganda)

Other issues emphasised during the radio talk shows included infection prevention and hygiene during which intervention recipients and other listeners were sensitised on why and how to wash their hands in their homes. For example, during these talk shows, listeners were asked to consider the needs of persons of short stature while setting up handwashing facilities in their homes.

#### Loss of Foreign, Commonwealth and Development Office funding

Given the challenging global operating environment precipitated by the COVID-19 pandemic, the United Kingdom Foreign, Commonwealth, & Development Office initiated funding cuts which affected the DIG project, implemented as part of a broader cut to a raft of its programming. The unexpected loss of funding at the midway stage of the programme implementation dealt a major blow to the implementation of the programme activities. Specifically, it affected the recruitment of rehabilitation personnel who were involved in the delivery of essential services such as physiotherapy and occupational therapy. This point was highlighted by one of the key informants:

‘At one point, we had to lay off physiotherapists yet we need their services. Of course, we later managed to lobby for some extra funding from BRAC-UK and we then brought back some of these staff but that was after some time. This certainly affected the quality and timing of our services.’ (BRAC Programme Officer, Kiryandongo district, Western Uganda)

Moreover, resourcing for other services was affected. Under the social protection pillar, a modest stipend was provided for 6 months to enable the intervention recipients pay for the basic necessities such as food, medical care and where necessary, shelter. Of great significance was the health subsidy provided under this pillar which was always provided based on the need and nature of healthcare required. This subsidy prioritised psychosocial support for project intervention recipients especially during the COVID-19 pandemic lockdown period – as explained by one of the key informants:

‘During the COVID-19 period, we had numerous issues to do with psychosocial and mental health issues. At some point, we realized that some intervention recipients were not able to access some drugs, so we had to lobby for them to continue receiving the stipend. Unfortunately, we suffered a setback when FCDO withdrew some funds and we were not able to meet some of the needs that were emerging on daily basis. But we pushed on and managed without necessarily losing intervention recipients to the pandemic.’ (BRAC Programme Officer, Nwoya district, Northern Uganda)

Intervention recipients of the DIG intervention were also aware of the challenges posed to the programme by COVID-19. Some reported that the project close-out was unsatisfactory, entailing poor provision of information about when the project would end, which they saw as related to the COVID-19 pandemic:

‘The end of the DIG-BRAC programme was a typical case of disappearing without alerting us (the intervention recipients at all). Even when rumours started circulating that they had gone, we failed to contact them. There was no news at all or any form of response. Of course, there was a time during the COVID-19 pandemic when word started circulating the programme would end but when some of us reached out to their staff for any information on this issue, they refused to answer until they disappeared.’ (Female beneficiary living with a physical disability, Gulu district, Northern Uganda)

Taken together, this shows how the DIG intervention experienced two detrimental challenges associated with the COVID-19 pandemic. The first was the public health and movement restrictions implemented by the Ugandan government as a means of infection control, while the second was the loss of funding to the programme by its United Kingdom donor, for which the COVID-19 pandemic was the primary proximal cause.

### Mechanisms of impact

#### Change in societal attitude towards persons with disabilities

According to respondents, the implementation of the DIG programme greatly contributed to a change in attitude towards persons with disabilities in the districts where its activities were carried out. Specifically, respondents perceived the sensitisation campaigns against long-held stigma towards such individuals as playing a significant role in changing minds and shaping knowledge and attitudes which in their view proved to be a key turning point in the way society perceived persons with disabilities. Furthermore, the empowerment and social protection measures designed and implemented under the project not only improved livelihoods, but also provided a firm basis for a new narrative on the way persons with disabilities can co-exist with the rest of society as explained by one of the key informants:

‘Prior to the implementation of the DIG programme, persons with disabilities were being looked at in the same category as minors who contributed nothing to their lives and society. Even they (the PWDs) also had a lot of self-stigma about themselves and were just there waiting for hand-outs. But when we went in with this programme, we witnessed an attitude change among the persons with disabilities and the communities where they reside. They became more involved in community activities, more active in their home activities and also took on the leadership roles at various levels. Generally, I would say that the DIG programme brought a positive attitude among persons with disabilities and changed lives.’ (BRAC Programme Officer, Gulu district, Northern Uganda)

Intervention recipients also shared positive views of the sensitisation campaigns conducted as part of the DIG intervention and the impact they had on stigma towards persons with disabilities:

‘Before this programme began the sensitisation of our communities to end stigmatising us for being disabled, I used not to be happy at all. And because my son with a physical disability was then seen by some of my family members as a curse to them. They would always abuse me that I am a bad woman and I have produced a lame child. You see this tribe, the Acholi people of Northern Uganda just talk even when you are hearing. They do not back-bite like the Bantu who can try to hide. These ones just talk. Someone comes home drunk and starts saying, “Aaa … you also … you have no child, he is lame and is going to die anytime”. But when the DIG programme awareness was intensified, the situation changed. They now look at my boy in a positive way and he feels loved too.’ (Female beneficiary living with a hearing impairment and mother to a 7-year-old son with a physical impairment in Oyam district, Northern Uganda)

The DIG programme efforts to end all forms of disability stigma also resulted into other consequences for intervention recipients such as building self-esteem as highlighted by a female respondent with a disability:

‘Prior to the sensitisation campaign, someone would call you and when you don’t respond because you have a hearing problem, they start abusing you. They would ask whether you are deaf and even shout at you. I would really feel low and useless in the community. But the DIG-BRAC programme staff showed love and respect. Overall, my self-esteem is quite high now.’ (Female beneficiary living with a hearing impairment, Nwoya district, Northern Uganda)

However, respondents highlighted that there were unintended attitudinal consequences of the DIG intervention also. Given the benefits provided by the programme to those who participated, a general feeling of jealousy emerged among some community members who envied what the persons with disabilities were either being given or had been empowered to do on their own. Over time, there were increased incidences of theft and burglary targeting the programme intervention recipients motivated by such jealousy as explained by one of the intervention recipients during an interview:

‘At first, the community didn’t bother so much about us but they started feeling bad when we began to reap the benefits of the DIG programme. They began targeting our properties and even lives because they felt that we were being pampered by BRAC. Even someone being taken for orthopedic review was looked at in a different way. Some community members felt that such persons are being favoured which led to thefts, house break-ins and personal attacks.’ (Male intervention recipient with disabilities, Kiryandongo district, Western Uganda)

The DIG programme implementation team responded to this issue by heightening their sensitisation campaigns to address the jealousy and stigmatisation issues. Additionally, psychosocial support was also intensified for intervention recipients who needed such services.

#### Social empowerment

Respondents who received the DIG intervention not only reported a change in societal attitudes towards disability, and associated improvements to self-esteem, but also positive changes in the way they interacted and engaged with the community. As one respondent explained:

‘The interventions helped us overcome poverty and graduate to the level of owning assets like cows, goats and pigs. We also started going to church because we had been socially empowered. We started mingling with the rest of the community members and accessing social services. We are also involved in politics and are demanding for our rights in society as granted by the national Constitution and other laws.’ (Female intervention recipient with disabilities, Oyam district)

Some intervention recipients also linked the practical support and skills training they had received through DIG to a broader mindset change about the future and the potential to be better off financially:

‘When the people from the DIG-BRAC programme came to us, they told us many things which helped change my mindset and I started seeing things differently. Because I was badly-off socially and financially at that time, they taught me many things such as, how to do business, how to take care of myself, how to eat well and how to do everything. And by the way it was not only me. It was for all programme intervention recipients – regardless of whether you have a disability or not. We all had a mindset change and I can say we visualize the future in a positive and completely different way!’ (Female beneficiary living with a physical disability in Kiryandongo district, Western Uganda)

‘During the financial management training, the DIG-BRAC project staff sensitised us on the importance of forming groups so that we could start saving some money. They taught us how to keep records of savings groups and this changed our minds on the way we keep records and manage our finances.’ (Male beneficiary living with visual impairment, Kiryandongo district, Western Uganda)

In many ways, the programme not only augmented the principle of social empowerment to its intervention recipients but also that of access to, ownership and management of assets in their lives. From the above-stated quotations, it is clearly evident that social empowerment did not only manifest itself in the self-perception that the intervention recipients felt that they could just do certain things such as participate in politics or attend church services (which meant a lot to some of them), but also made them realise that they can actually access, own and manage key assets in their lives such as animals and forms of wealth. This was further emphasised by another beneficiary who added:

‘During the mapping exercise, I remember people could say to me: I am a person with disability, that we would not benefit much from the programme. Even after starting to implement the activities, many of us never believed that much will change. After all, we had seen many other initiatives in the past. But with time, we could see tangible benefits which we didn’t expect. A person with disabilities who, for instance, used to think that they cannot take care of a goat is now able to care of their own goats. Here in Gulu district, where a person was neglected and put in her own space, [she] was rehabilitated and is now able to sit in a wheelchair and get money by herself. She no longer has to depend on other people all the time for livelihoods … She can do all that on her own and also take care of her on assets.’ (Female intervention recipient with disabilities, Gulu district, Western Uganda)

From the foregoing findings, it is clearly evident that there is an intersection between the socio-economic and political empowerment attained by the programme intervention recipients. The programme implementation team specifically focussed on providing tangible resources such as animals and wheelchairs to selected intervention recipients in order to empower them to participate in income generating activities. This form of empowerment contributed to helping to change mindsets, and respondents were able to participate in social activities in the church and political spheres which had not happened before the programme was implemented. Overall, it is part of the indirect benefits narrative that emerged through this evaluation and forms part of its success story.

## Discussion

This qualitative process evaluation has summarised the structures and resources used to deliver the intervention and identified key contextual influences and mechanisms through which the intervention exerted its influence. Specifically, the process evaluation identified the COVID-19 pandemic and loss of FCDO funding as salient contextual factors. Changes in societal attitudes and perceived increases in social empowerment were identified as important mechanisms of impact.

This qualitative process evaluation complements existing quantitative studies of the DIG programme. Analysis of the DIG programme’s impact has identified that the intervention significantly increased the economic wellbeing of households in the intervention group, relative to the control (Kipchumba et al. forthcoming). Moreover, a complementary quantitative process evaluation of the DIG programme found that disability-targeted components of the intervention like physiotherapy and rehabilitation were less important to achieving the desired outcomes of the programme, such as economic wellbeing, relative to other intervention components (Mapuwei et al. forthcoming). The present qualitative process evaluation complements these findings by highlighting the detrimental roles that COVID-19-related factors played on intervention provision. It is notable that participants in our study drew a distinction between the impact of national infection prevention and control measures, such as restrictions on the movement of individuals, and the loss of FCDO funding on intervention activities. In particular, participants highlighted that these challenges disrupted the provision of face-to-face activities to intervention recipients, such as physiotherapy, providing a possible explanation as to why provision of disability-targeted activities like physiotherapy and rehabilitation had smaller effects relative to other DIG components. While participants highlighted a detrimental impact of national infection prevention and control measures, such as how many staff could travel in a car together, the programme was still able to deliver intervention activities, albeit at a reduced rate. However, when funding was lost because of the impact of COVID-19, the programme had to discontinue some activities completely such as physiotherapy and psychosocial support until an alternative source of funding was found. This highlights the importance of maintaining funding sources amid national challenges and crises such as COVID-19. Notwithstanding, it is encouraging that the DIG programme had a positive impact on economic wellbeing, even amid the challenging circumstances of COVID-19.

This study identified a change in societal attitude towards persons with disabilities and social empowerment as important mechanisms through which the intervention exerted impact according to implementers and DIG participants. This was consistent with the DIG Theory of Change. Respondents perceived that the sensitisation campaigns held under the DIG programme brought about attitudinal change, both in terms of internal attitudes (i.e., self-stigma and mindset) and attitudes held about disability by the community, which they perceived as contributing to economic wellbeing. This may lead to a virtuous cycle, as participants are seen to do more and become more accepted as a result; alternatively, the attitudinal change may be short-lived and dissipate. Quantitative analysis of the attitudinal impacts of the DIG programme is forthcoming. In relation, some intervention recipients also highlighted unintended consequences related to attitudes, namely that feelings of jealousy were provoked among some community members who did not get the intervention, towards those that did. In some cases, this resulted in theft and personal attacks targeted towards the DIG recipients. This highlights the need for future iterations of the DIG programme to consider how to more effectively sensitise community members around intervention recipients, in order to ensure no beneficiary experiences a negative consequence.

This qualitative process evaluation adds to existing knowledge about the DIG intervention by shedding light on its contextual factors and mechanisms of impact. However, the study had some limitations. Notably, in order to gain information about the implementation process (i.e., structures and resources), mechanisms of impact and contextual influences, the research has relied primarily upon information provided by desk reports published directly by the intervention implementers. While these data contain insights relevant to understanding the intervention, for example, in terms of how many participants received individual activities, these data are not independent observations of the intervention. Monitoring and evaluation data also could not be obtained from implementers. Thus, this research study has not attempted to draw independent evaluative conclusions about what participants received from the intervention (e.g. implementation fidelity and dose; see Moore et al. 2015). Instead, it describes what the DIG programme itself has reported about implementation. Moreover, although the research includes individuals with a diverse range of impairment types, the study did not include persons with psychiatric or psychosocial disabilities. Nevertheless, our findings provide important new insights about how the DIG programme worked and how the context it was implemented in influenced intervention delivery and impact.
